# Identification of a New Antifungal Peptide W1 From a Marine *Bacillus amyloliquefaciens* Reveals Its Potential in Controlling Fungal Plant Diseases

**DOI:** 10.3389/fmicb.2022.922454

**Published:** 2022-06-13

**Authors:** Qiao Wen, Ruizhe Liu, Zhenxiao Ouyang, Tianliang He, Weini Zhang, Xinhua Chen

**Affiliations:** ^1^Key Laboratory of Marine Biotechnology of Fujian Province, Institute of Oceanology, College of Marine Sciences, Fujian Agriculture and Forestry University, Fuzhou, China; ^2^Southern Marine Science and Engineering Guangdong Laboratory, Zhuhai, China

**Keywords:** plant pathogenic fungi, *Bacillus amyloliquefaciens*, antifungal peptide, antifungal activity, Arctic Ocean

## Abstract

A bacterium, *Bacillus amyloliquefaciens* W0101, isolated from the Arctic Ocean, showed potent antifungal activity against several plant pathogenic fungi. An antifungal peptide W1, with a molecular weight of approximately 2.4 kDa, was purified from the culture supernatant of the strain W0101 using ion-exchange chromatography and high-performance liquid chromatography. By analysis of Liquid Chromatograph-Mass Spectrometer, the peptide W1 was identified as a new antifungal peptide derived from the fragment of preprotein translocase subunit YajC. Further analysis revealed that W1 could disrupt the hyphae and spores of *Sclerotinia sclerotiorum* and inhibit its growth. W1 suppressed *S. sclerotiorum and Fusarium oxysporum* at a minimum inhibitory concentration of 140 and 58 μg/ml, respectively. The antifungal activity of W1 remained stable at 20–80°C or pH 6–11, with reduced activity at 100–110°C and pH 4–5, and under three protease treatments. Additionally, W1 also had a certain extent of metal ion resistance. These results therefore suggest that the peptide W1 from marine *B. amyloliquefaciens* W0101 may represent a new antifungal peptide with potential application in the biocontrol of plant diseases.

## Introduction

Fungal diseases have always been one of the main restricting factors affecting the good quality and high yield of crops (Montesinos, 2007). According to statistics, about 80% of plant diseases are caused by fungal infection (Someya, 2008). Fungal pathogens, such as *Fusarium oxysporum*, *Alternaria longipes*, and *Magnaporthe grisea*, generally cause necrosis, rot, and wilting in leaves, stems, or fruits of plants, decreasing crop yield and quality (Talbot, 2003; Ajilogba and Babalola, 2013; Shoaib et al., 2014). Moreover, some pathogenic fungi can even produce a variety of mycotoxins and metabolites that are harmful to humans and animals, posing a great threat to the safety of agricultural products (Hussein and Brasel, 2001). Till present, the utilization of chemical fungicides has been the main approach that controls fungal infections (Kumar and Kumar, 2019). It is estimated that around 3 billion kilograms of agricultural chemicals are applied in the agricultural industry each year. However, the long-term abuse of chemical fungicides in agriculture has resulted in a gradual increase in fungal resistance to chemical fungicides, a detrimental effect on the environment, and a health threat to humans and animals (Sharma et al., 2020). On account of the harmful effects of chemical germicides, ecologically safe and cost-effective measures as an alternative to chemical control are urgently needed to achieve the sustainable development of agriculture (Fisher et al., 2018).

Antifungal peptides (AFPs) are a kind of defensive peptide with antifungal activity that exists widely in plants, animals, and microorganisms (Yan et al., 2015). AFPs generally contain 10–100 amino acid residues with a molecular weight of less than 10 kDa and play an important role in the defense against fungal infection in plants (Montesinos, 2007; Yan et al., 2015). The special cationic and amphiphilic structures make AFPs kill fungal cells by inhibiting the synthesis of fungal cell walls and interacting with components in the membrane and intracellular targets (Li et al., 2021). These special mechanisms, different from chemical fungicides, are considered to be the effective ways to reduce the development of drug resistance (Dhanasekaran et al., 2012). Thus, AFPs may become a new generation of promising antifungal agents in future anti-infectious applications.

During the past few decades, *Bacillus* species have been widely used in the control of crop diseases (Ongena and Jacques, 2008). *B. subtilis* and its metabolites were used to control blue mold on citrus (Waewthongrak et al., 2015). *Bacillus cereus* and *Bacillus safensis* had a significant effect in preventing leaf spot and blight disease (Roy et al., 2018). *Bacillus amyloliquefaciens* was also found to have potential antifungal activity against the pathogens of root rot disease and black rot disease in sweet potatoes (Wang et al., 2020). Several strains of *Bacillus* were also found to produce AFPs against *Aspergillus niger* (*B. amyloliquefaciens* BH072), *Alternaria solani* (*Bacillus marinus* B-9987), *Beauveria bassiana* (*B. amyloliquefaciens* SWB16), *F. oxysporum* (*B. cereus* QQ 308), *Fusarium solani* (*B. cereus* QQ 308), and *Pythium ultimum* (*B. cereus* QQ 308; Chang et al., 2007; Gao et al., 2009; Zhao et al., 2013; Wang et al., 2014). Recently, researchers have isolated several antifungal microbes from the polar regions, such as *Bacillus sp*. ANT_WA51, *B. amyloliquefaciens* PC3 and *Penicillium chrysogenum* A096, which produce active antifungal peptides and show potential application values in agriculture (Chen et al., 2013; Cui et al., 2017; Ding et al., 2018, 2019; Styczynski et al., 2022). Therefore, screening for antifungal bacteria from the polar regions has become a new approach to controlling agricultural diseases (Núñez-Montero and Barrientos, 2018). In this study, we isolated a strain of *B. amyloliquefaciens* W0101 with remarkable antifungal activity from a sediment sample of the Arctic Ocean. An antifungal peptide W1 was purified and identified from the culture supernatant of the strain W0101. We then investigated the mechanisms underlying W1 inhibition of *S. sclerotiorum* and also analyzed the physiochemical properties of W1.

## Materials and Methods

### Tested Strains

The tested fungus *Paecilomyces variotii* (CGMCC 3.776) was provided by the China General Microbiological Culture Collection Center, and *F. oxysporum* (ACCC 31352), *S. sclerotiorum* (ACCC 36081), *Rhizoctonia solani* Kühn (ACCC 36316), *A. longipes* (ACCC 30002), *Colletotrichum gloeosporioides* (ACCC 31200), *Alternaria gaisen* (ACCC 37473), *Botryosphaeria dothidea* (ACCC 38026), and *Phomopsis amygdali* (ACCC 37078) were provided by Agricultural Cultural Collection of China. All fungi were grown on potato dextrose agar (PDA, Haibo Biotechnology Company, China) plates at 30°C.

### Isolation of Antifungal Strains

Sediment sample was collected from the Arctic Ocean (168°09′41″W, 69°13′3″7) during the sixth Chinese National Arctic Expedition. The sediment sample was cultured by oscillation with sterile seawater and glass beads at 4°C for 6 h. An aliquot of supernatant liquid homogenized mixture was put into centrifuge tubes containing sterile seawater and a serial 10-fold dilution was done up to 10^–6^. Approximately 100 μl of the diluted samples were spread on 2216E agar media (Haibo Biotechnology Company, Qingdao, China) in each dilution. Later, all plates were incubated at 28°C for 48 h. The strains were selected based on their morphological features and inoculated into LB medium for further growth to evaluate their antifungal potential.

### Antifungal Activity Detection

The disk diffusion method was applied in the determination of antifungal protein produced by strain W0101 (Chen et al., 2013; Ding et al., 2018, 2019) In brief, a hyphal block of tested fungus was inoculated onto the center of PDA plates, and 60 μl of the culture supernatant of strain W0101, purified or synthesized peptide in PBS buffer, was added to symmetrical sites 20 mm away from the pathogen. The plates were cultured for 48 h at 30°C to observe for antifungal activity. The sample treatment was repeated in triplicate.

### Identification of Strain W0101

Genomic DNA of strain W0101 was extracted using an E.Z.N.A. Bacterial DNA Kit according to the manufacturer’s protocol (Omege Bio-Tek, Guangzhou, China). The 16S rDNA sequence of strain W0101 was amplified using universal primers (27F and 1492R), sequenced by Sangon Biotech (Shanghai, China), and aligned with NCBI rRNA/ITS databases using the blastn algorithm.^1^

For phylogenetic analysis, the 16S rDNA sequences were aligned using the MAFFT program with default parameters (Katoh et al., 2002). A phylogenetic tree was constructed with the maximum likelihood estimate method using the MEGA 7.0 software (Kumar et al., 2016). The tree reliability was assessed by the bootstrap method based on 1,000 replicates.

The genome sequencing of strain W0101 was performed using PacBio and Illumina Miseq sequencing by Majorbio Biotech Co., Ltd. (Shanghai, China). The methods of genome assembling for the strain W0101 genome refer to our previous study (Li et al., 2020). An average nucleotide identity (ANI) analysis was performed among the genomes of the strain W0101 and other 8 *Bacillus* strains using Jspecies (version 1.2.1; Richter and Rossello-Mora, 2009).

### Isolation and Purification of Antifungal Peptides From W0101

For the isolation and purification of antifungal peptides, refer to our previously published methods (Chen et al., 2013; Wen et al., 2014; Cui et al., 2017; Wu et al., 2018). The stain W0101 was cultured in LB medium at 28°C for 48 h before centrifugation at 8,000 × *g* for 10 min at 4°C. The supernatant was filtered using a 0.45-μm membrane and respectively added ammonium sulfate with six saturation concentrations (0, 20, 40, 60, 80, and 100%) to let stand overnight at 4°C. The crude proteins were collected after centrifugation at 12,000 × *g* for 20 min at 4°C. Then, it was dissolved in a suitable volume of 20 mM Tris–HCl buffer (pH 8.07). After being dialyzed with the same buffer for 48 h at 4°C, the crude protein was concentrated by freeze-dying.

The crude protein sample was separated by ion exchange chromatography with an AKTA Pure 25 system. The sample solution was loaded onto a HiTrap DEAE Sepharose Fast Flow column, which was pre-equilibrated with starting buffer A (20 mM Tris–HCl, pH 8.07) for the primary purification step. The elution procedure was as follows: 0% elution buffer B (20 mM Tris–HCl, 1 M NaCl, pH 8.07), 5 CV (column volume); 25% elution buffer B, 4 CV; 50% elution buffer B, 3 CV; and 100% elution buffer B, 5 CV. Each fraction was collected according to the absorbance of 280 nm. Then, all fractions were concentrated using ultrafiltration and determined their antifungal activity. The antifungal fraction was purified by a HiTrap Q Sepharose Fast Flow column, which was pre-equilibrated with starting buffer C (20 mM piperazine, 400 mM NaCl, pH 9.73). The elution procedure was carried out as follows: 13–34% elution buffer D (20 mM piperazine, 1 M NaCl, pH 9.73), 24 CV; 80% elution buffer D, 5 CV; and 100% elution buffer D, 5 CV. Finally, the purity of antifungal components from the HiTrap Q column was detected by reversed phase-high performance liquid chromatography (RP-HPLC) using Waters Alliance E2695 equipped with an analytic C18 reverse-phase column at a flow rate of 1 ml/min, measured at 280 nm. Solvent A (0.1% (w/v) trifluoroacetic acid (TFA, Sigma-Aldrich, St. Louis, United States) in water and solvent B (0.1% TFA in acetonitrile) were used as the mobile phases in a gradient elution mode (5–90% solvent B, 50 min). All the peaks were collected. The purity of the corresponding compounds was evaluated by sodium dodecyl sulfate-polyacrylamide gel electrophoresis (SDS-PAGE), and their antifungal activity was determined.

### Identification of Antifungal Proteins by Liquid Chromatograph-Mass Spectrometer

The purified antifungal peptide was identified at Beijing Bio-Tech Pack Technology Company Ltd. (Beijing, China). The samples were first subjected to trypsin digestion. Liquid Chromatograph-Mass Spectrometer (LC-MS/MS) analysis of the digested samples was performed on an Ultimate 3000 HPLC system (Thermo Fisher Scientific, Waltham, United States) interfaced online to an Orbitrap Fusion™ Lumos™ Tribrid™ Mass Spectrometer (Thermo Fisher Scientific). The mobile phase was composed of 2% acetonitrile with 1% formic acid (solvent A) and 80% acetonitrile with 0.1% formic acid (solvent B). Both mobile phases were degassed for 30 min in a sonicator bath. 5 μl of sample was first loaded onto a trapping column packaged with PepMap RPLC C_18_ (5 mm × 300 μm i.d., 5 μm) at a flow rate of 10 μl/min. After 2 min, the sample was separated on a nanocolumn column packaged with PepMap RPLC C_18_ (150 mm × 150 μm i.d., 1.9 μm) at a flow rate of 200 nl/min using a solvent A and B mixture. The mobile phase gradient was as follows: 4–10% of solvent B in 5 min, 10–22% in 80 min, 22–40% in 25 min, 40–95% in 5 min, and 95% during 5 min.

Subsequently, the sample was infused into the mass spectrometer *via* a dynamic nanospray probe (Thermo Fisher Scientific) and analyzed in positive mode. The parameters of primary mass spectrometry are as follows: resolution, 70,000; AGC target, 3e6; maximum IT, 60 ms; scan range, 300–140 m/z. The parameters of secondary mass spectrometry are as follows: resolution, 17,500; AGC target, 5e4; maximum IT, 15 ms; TopN, 5; scan range, 200–2,000 m/z.

The raw mass data were analyzed using MaxQuant version 1.6.2.10 (Tyanova et al., 2016) to match the protein data of *B. amyloliquefaciens* W0101 (GenBank accession no. NZ_CP090477), whose genome was sequenced in this study. The analysis settings were as follows: fixed modifications, carbamidomethyl (C); variable modifications, oxidation (M) and acetyl (protein N-term); enzyme, trypsin; maximum missed cleavages, 2; peptide mass tolerance, 20 ppm; fragment mass tolerance, 20 ppm; mass values, monoisotopic; significant threshold, 0.01.

### Thermostability Assay of W1

To evaluate the thermostability of W1, the purified peptide with a concentration of 160 μg/ml was incubated at 20, 40, 60, 80, 100, 110, and 121°C (autoclaved) for 20 min. After cooling to room temperature, the residual antifungal activity of peptide W1 was tested against *F. oxysporum* using the agar diffusion bioassay, which was described above. The peptide W1 without heat treatment and piperazine buffer (20 mM, pH 9.73) were used as the positive and blank controls, respectively. All assays were repeated three times independently.

### pH Stability Test of W1

The purified peptide W1 with a concentration of 160 μg/ml was exposed to a pH range from pH 3.0 to pH 11.0 using 50 mM glycine-HCl (pH 3.0), 50 mM sodium acetate (pH 4.0), 50 mM Na_2_HPO_4_-citric acid (pH 5.0 and 6.0), 50 mM Na_2_HPO_4_-NaH_2_PO_4_ (pH 7.0), 20 mM Tris–HCl (pH 8.0), 50 mM glycine-NaOH (pH 9.0), 20 mM piperazine (pH 10.0), and 50 mM Na_2_HPO_4_-NaOH (pH 11.0). After incubation for 2 h at room temperature, the antifungal activity against *F. oxysporum* of the reaction mixture were tested with all buffer solution as the blank controls. All assays were repeated three times independently.

### Effect of Metal Ions on the Antifungal Activity of W1

To evaluate the effect of metal ions on the antifungal activity of W1, we selected several common metal ions such as Mg^2+^, Ca^2+^, Mn^2+^, Ba^2+^, Na^+^, K^+^, Fe^2+^, and Cu^2+^, which were dissolved in 20 mM piperazine buffer (pH 9.73). The purified peptide W1 with a concentration of 160 μg/ml was treated with different ions solutions at the final concentrations of 20 mM Mg^2+^, Ca^2+^, Mn^2+^, Ba^2+^, Na^+^, and K^+^ and 6 mM Fe^2+^ and Cu^2+^, respectively, at room temperature for 3 h. The antifungal activity against *F. oxysporum* of the reaction mixture was subsequently determined. The peptide W1 without ion solution treatment was used as the positive control, and ion solutions were used as blank controls. All assays were repeated three times independently.

### Effect of Proteases on the Antifungal Activity of W1

To detect the sensitivity of W1 to proteases, the purified peptide with a concentration of 160 μg/ml was subjected to treatment with 1 mg/ml proteinase K (Sigma-Aldrich, United States) at 58°C for 2 h, or 1 mg/ml papain (Sigma-Aldrich, United States) at 55°C for 2 h, or 1 mg/ml trypsin (Sigma-Aldrich, United States) at 37°C for 2 h. The purified W1 treated under the same conditions but without proteases was used as control. All assays were repeated three times independently.

### MIC Determination of W1

The minimum inhibitory concentration (MIC) of W1 against *F. oxysporum* and *S. sclerotiorum* was determined using the paper disk diffusion method (Wen et al., 2014; Rao et al., 2015). In brief, the purified W1 was diluted into 20 mM piperazine buffer (pH 9.73) with final concentrations of 0.464, 0.232, 0.116, 0.058, and 0.029 μg/μl, respectively. On paper disks placed 2 cm from the hyphal margin of fungus on a PDA plate, 60 μl of serially diluted samples were added. Later, the plate was incubated at 30°C for 24–48 h. The MIC was determined as the lowest concentration of W1 that could inhibit visible mold growth and was calculated as the total peptide concentration added to each paper disk (microgram per disk). The piperazine buffer (20 mM) was used as the blank control. All assays were repeated three times independently.

### Scanning Electron Microscope Analysis

To analyze the antifungal mechanism of W1 against pathogenic fungi, 1 ml of PDA medium containing mycelium of *F. oxysporum* or *S. sclerotiorum* was poured on the surface of an aluminum specimen that is used for Scanning Electron Microscope (SEM) and incubated at 30°C for 72 h. After incubation, 20 μl of the purified W1 (160 μg/ml) was added on the surface of the aluminum specimen and continued to incubate at 30°C for 24 h. The PBS solution as control was used to perform the same process. Each specimen was washed in glutaraldehyde solution (1 mM NaH_2_PO_4_-Na_2_HPO_4_, 2.5% glutaraldehyde, pH 7.4) and finally fixed in glutaraldehyde solution at 4°C for 12 h. After fixation, the specimens were washed with phosphate solution (1 mM NaH_2_PO_4_-Na_2_HPO_4_, pH 7.4) three times for 15 min. The dehydration was performed using a series of acetones (35, 50, 75, 95, and 100%) for 10 min, respectively. Each step of the dehydration was repeated three times. Then, the specimens were critically dried for 90 min and coated with gold in a sputter coater. Images were observed and captured using a JSM-6380LV SEM (JEOL Instruments Inc., Tokyo, Japan) at 15 kV.

### Statistical Analysis

Statistical analyses were performed using Origin. Data are shown as the mean ± standard deviation (SD), and the results were from at least three experiments. Analysis of significance (ANOVA) followed by the Duncan multiple ranges test was applied using the SPSS software to determine the significant differences at 99% confidence intervals. Data were considered to be statistically different when *p < 0.05, ^**^p < 0.01, and ^***^p < 0.001.

### Bioinformatics Analysis

The molecular weight (MW) and theoretical isoelectric points (pI) of W1 were calculated by the Expert Protein Analysis System (ExPASy; ProParam^2^). The secondary structure was analyzed using online bioinformatics tools at the website of NovoPro.^3^ The three-dimensional structure prediction of a peptide was performed using PEP-FOLD3.^4^

## Results

### Identification of the Strain W0101 With Antifungal Activity

**FIGURE 1:**
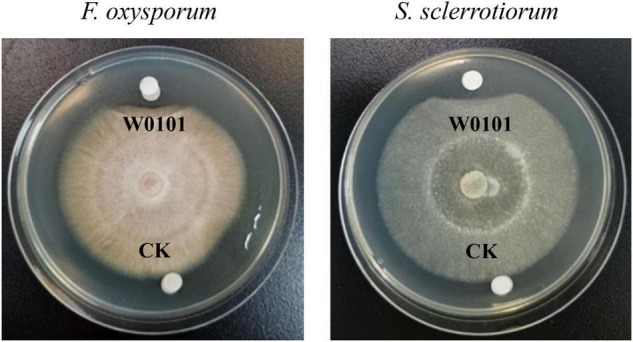
The antifungal activity of the strain W0101 against *Fusarium oxysporum* and *Sclerotinia sclerotiorum*. W0101, culture supernatant of W0101; CK, LB medium as control.

An antifungal strain W0101 was isolated from a sediment sample (168°09′41″W, 69°13′37″N) collected from the Arctic Ocean during the sixth Chinese National Arctic Expedition. The fermentation supernatants of W0101 culture showed obvious inhibitory activity against 9 plant pathogenic fungi, including *A. longipes*, *A. gaisen*, *B. dothidea*, *C. gloeosporioides*, *F. oxysporum*, *P. variotii*, *P. amygdali*, *R. solani* Kühn, and *S. sclerotiorum* (Figure 1 and Supplementary Figure 1). The diameters of the inhibition zone ranged from 17 to 34 mm (Supplementary Table 1).

**FIGURE 2:**
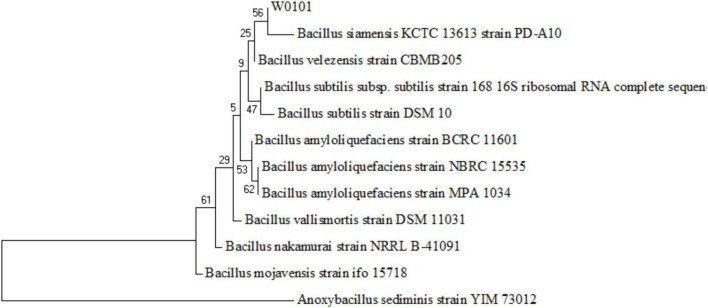
Phylogenetic tree of the strain W0101 based on the 16S rRNA gene sequences. The tree is constructed with the maximum likelihood estimate method by the MEGA 7 software. The numbers on the nodules represent frequency of 1,000 bootstrap replicates.

The 16S rDNA sequence of the strain W0101 (NZ_CP090477, locus_tag = LXM91_00040) showed the highest identity of 99.651, 99.65, and 99.65% with that of *Bacillus velezensis* strain CBMB205 (NR_116240.1), *B. amyloliquefaciens* strain NBRC 15535 (NR_041455.1), and *B. amyloliquefaciens* strain MPA 1034 (NR_117946.1), respectively (Supplementary Table 2). The phylogenetic tree based on the 16S rDNA sequences also showed that the strain W0101 gathered into one branch with *Bacillus siamensis* strain PD-A10 and *B. velezensis* strain CBMB205 (Figure 2). These results indicated that the strain W0101 was classified as *Bacillus*.

To further confirm the taxonomy of the strain W0101, the complete genome of the strain W0101 was sequenced (Accession no. NZ_CP090477) and used to perform the analysis of average nucleotide identity (ANI). The ANIm value between the strain W0101 and *B. amyloliquefaciens* strain B15 (NZ_CP014783.1) was up to 99.14%, suggesting that the strain W0101 was a *B. amyloliquefaciens* isolate from the Arctic Ocean (Table 1).

**TABLE 1:** OrthoANI analysis for the strain W0101.

Bacterial strains	Accession no.	ANIm (%)
*Bacillus amyloliquefaciens* strain B15	NZ_CP014783.1	99.1423
*Bacillus velezensis* strain BIM B-439D	NZ_CP032144.1	98.7148
*Bacillus vallismortis* strain NBIF-001	NZ_CP020893.1	98.2246
*Bacillus velezensis* strain YB-130	NZ_CP054562.1	98.2184
*Bacillus amyloliquefaciens* strain MBE1283	NZ_CP013727.1	97.8043
*Bacillus velezensis* strain FJAT-45028	NZ_CP047157.1	97.7934
*Bacillus amyloliquefaciens* strain DH8030	NZ_CP041770.1	97.7633
*Bacillus atrophaeus* strain BA59	NZ_CP024051.1	77.6409

### Purification of an Antifungal Peptide

**FIGURE 3:**
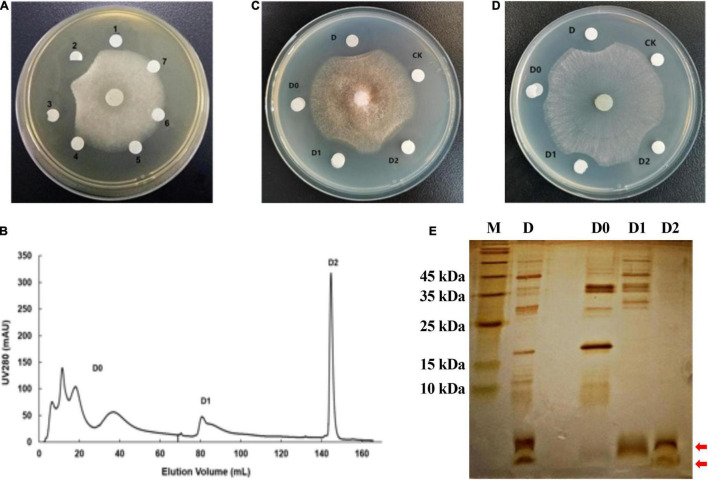
Purification of antifungal protein by DEAE Sepharose Fast Flow column. **(A)** The antifungal activity of crude proteins salted out at six concentrations of ammonium sulfate against *F. oxysporum*. The numbers 1–7 represent the crude proteins salted out at 0, 20, 40, 60, 80, and 100% of ammonium sulfate, respectively. The number 7 represents the fermentation supernatant without protein. **(B)** Chromatographic profile of crude proteins of the strain W0101 extract on HiTrap DEAE Sepharose Fast Flow column using AKTA pure 25 system. **(C)** The antifungal activity of several fractions from crude protein against *F. oxysporum.* **(D)** The antifungal activity of several fractions from crude protein against *S. sclerotiorum*. **(E)** 15% SDS-PAGE profile of several fractions in the separation of crude proteins. The red arrows show the two distinct protein bands of fraction D2.

The antifungal protein was extracted from the fermentation supernatants of the strain W0101 by salting out at 40% saturation of ammonium sulfate. The crude protein exhibited the strongest antifungal activity against *F. oxysporum* (Figure 3A), and was further purified using a two-step ion exchange chromatography. After loading on the DEAE Sepharose Fast Flow column, the crude protein was isolated into three major fractions (D0, D1, and D2; Figure 3B), of which fraction D2 was found to possess mighty antimicrobial activity against *F. oxysporum* and *S. sclerotiorum* (Figures 3C,D). The result of SDS-PAGE showed that the fraction D2 contained two distinct protein bands with a molecular mass of less than 10 kDa (Figure 3E).

**FIGURE 4:**
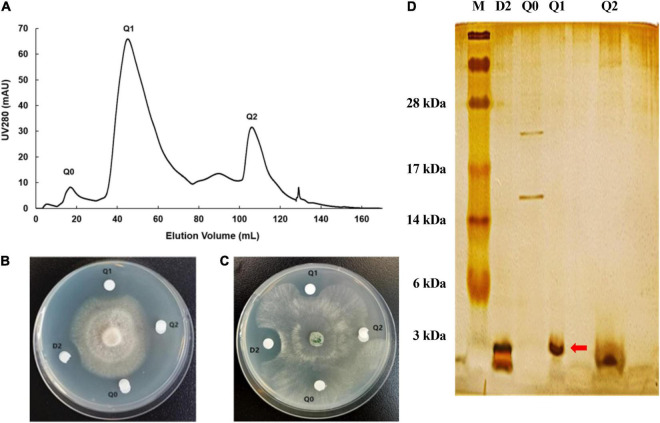
Purification of antifungal protein by Q Sepharose Fast Flow column. **(A)** Chromatographic profile of D2 from DEAE separation on HiTrap Q Sepharose Fast Flow column using AKTA pure 25 system. **(B)** The antifungal activity of several fractions from component D2 against *F. oxysporum.* **(C)** The antifungal activity of several fractions from D2 against *S. sclerotiorum*. **(D)** 15% SDS-PAGE profile of several fractions from Q2. The red arrow shows the distinct protein band of component Q1.

Subsequently, three major components (Q0, Q1 and Q2) were separated from the bioactive fraction D2 with the Q Sepharose Fast Flow column (Figure 4A), and component Q1 showed antimicrobial activity against *F. oxysporum* and *S. sclerotiorum* by plate inhibition test (Figures 4B,C). Component Q1 contained only a single protein band with a molecular weight of less than 3 kDa on the SDS-PAGE, corresponding to one of the two bands of fraction D2 (Figure 4D). Finally, the antifungal component Q1 was analyzed on a RP-HPLC, and a unique peak appeared at a retention time of 27.933 min compared with the standard of the buffer solution (Supplementary Figure 2).

### Mass Spectrometry Analysis and Identification of W1

**FIGURE 5:**
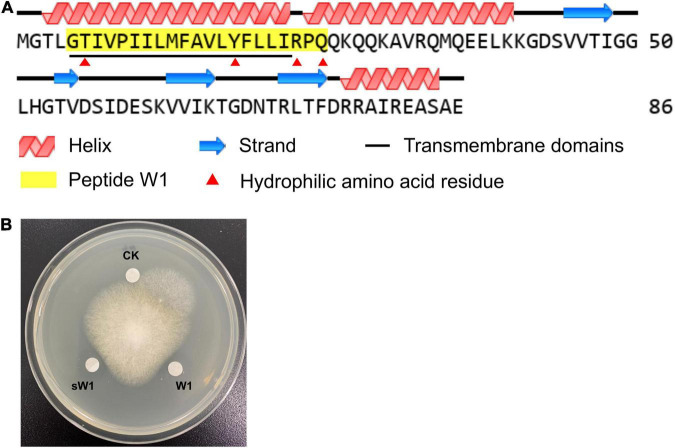
The structure and antifungal activity of W1. **(A)** Protein structure analysis of YajC and W1. The GenBank database reference number of YajC was UOI89425.1. **(B)** The antifungal activity of purified peptide (W1) and synthesized peptide (sW1) against *F. oxysporum*.

By LC-MS/MS analysis, W1 was identified as a peptide composed of 21 amino acid residues. This peptide completely matched a partial N-terminal sequence (G5 to Q26) of preprotein translocase subunit YajC of *B. amyloliquefaciens* W0101 (GenBank accession no. UOI89425.1; Table 2 and Figure 5A). The speculated molecular weight of W1 was 2,416.432 Da, consistent with the result of W1 electrophoresis (Figure 4D). The secondary structure analysis showed that W1 was a helical peptide and rich in hydrophobic amino acid residues, but contained a cationic, hydrophilic arginine in the C-terminal (Figure 5A). The three-dimensional structure of W1 predicted using PEP-FOLD3 also showed a helical structure, which was in agreement with the result of the secondary structure analysis (Supplementary Figure 3). These results indicated that W1 might be a peptide fragment cleaved from the YajC protein.

**TABLE 2:** Mass spectrum identification of W1.

Peptide sequence	Length	Mass (Da)	Matched protein	Protein ID in database*	Unique sequence coverage (%)	Score
GTIVPIILMFAVLYFLLIRPQ	21	2416.432	YajC	UOI89425.1	24.40%	128.97

To further identify W1, we artificially synthesized W1 (sW1). As shown in Figure 5B, the synthesized peptide showed significant antifungal activity compared with the control group, which was lower than that of purified peptide W1.

### Antifungal Mechanism of W1

**FIGURE 6:**
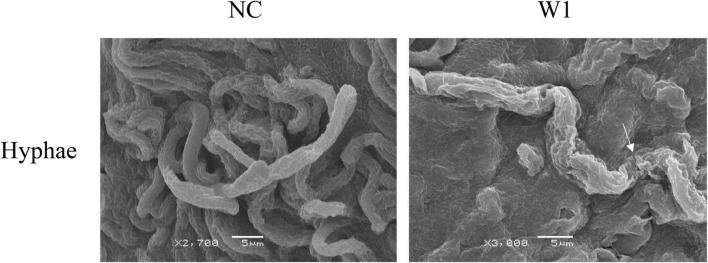
Scanning electron microscopy observation of hyphae of *F. oxysporum* treated with W1. The mycelium of *F. oxysporum* was treated with the purified W1 (160 μg/ml) at 30°C for 24 h, while treated with PBS as control. Images was observed and captured using a JSM-6380LV SEM (JEOL Instruments Inc., Japan) at 15 kV. The white arrows represent the morphological deformation in hyphae when treated with W1.

To investigate the antifungal mechanism of W1 on plant pathogenic fungi, scanning electron microscopy (SEM) was used to examine the effect of W1 on the morphology of the mycelium of *F. oxysporum*. After W1 treatment, the cell surface of hyphae was highly roughened and shriveled, while untreated hyphae cells were intact and smooth (Figure 6). Similar morphological changes of hyphae were also observed in *S. sclerotiorum* treated with W1 (Supplementary Figure 4). These results suggest that W1 could destroy the cell walls of hyphae of pathogenic fungi.

### Physiochemical Properties of Antifungal Protein W1

**FIGURE 7:**
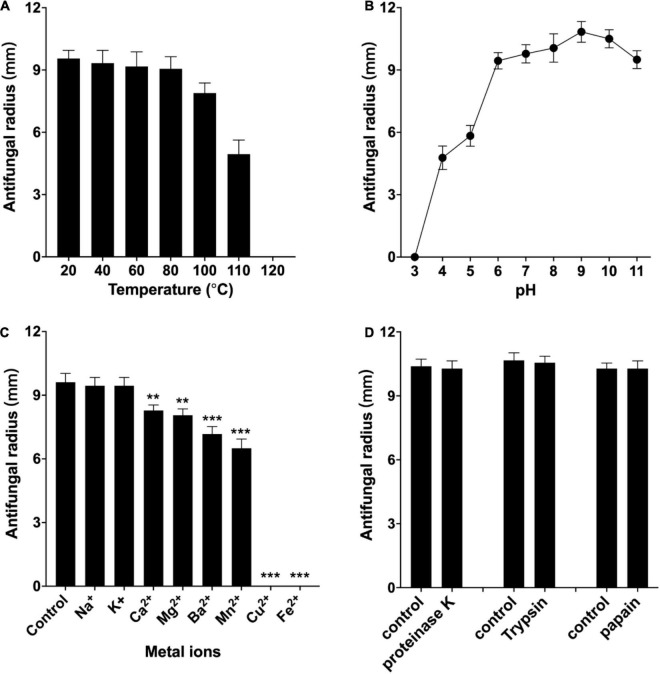
Effect of temperature **(A)**, pH **(B)**, metal ions **(C)**, and proteases **(D)** on antifungal activity of the purified peptide W1 against *F. oxysporum*. The purified W1 with a concentration of 160 μg/ml was used in all assays, which were repeated three times independently. The results are presented as mean ± SD, and representative images are shown. **p < 0.01, ***p < 0.001 vs. normal group.

The antifungal activity of the purified W1 against *F. oxysporum* was decreased by less than 10% when incubating at 40, 60, and 80°C for 20 min, whereas its antifungal activity was approximately retained at 75, 50%, and completely lost after incubating at 100, 110, and 121°C for 20 min, respectively (Figure 7A). W1 also had a higher antifungal ability against *F. oxysporum* at pH 6–11, but its antifungal activity was significantly weakened at pH 4.0 and pH 5.0 and rapidly lost at pH 3.0 (Figure 7B). The highest antibacterial activity of W1 protein against *F. oxysporum* was found to be at pH 9.0. Moreover, the antifungal activity of the purified W1 was not influenced by 20 mM Na^+^ and K^+^ and retained at least 70% with 20 mM Mg^2+^, Ca^2+^, Mn^2+^, and Ba^2+^. However, the antifungal activity was completely inhibited by 6 mM Fe^2+^ and Cu^2+^ (Figure 7C). It was also found that 1 mg/ml proteinase K, trypsin, and papain all showed no inhibitory effect on the antifungal activity of W1 (Figure 7D). Finally, the MICs of purified W1 against *F. oxysporum* and *S. sclerotiorum* were determined to be 58 and 140 μg/ml, respectively.

## Discussion

Fungal diseases have been posing a great threat to the agricultural industry. With the increasingly strict regulation of chemical fungicides, antifungal microorganisms and their metabolites are considered more and more important for controlling plant fungal diseases. In this study, a bacterial strain W0101 isolated from a sediment sample in the Arctic Ocean showed significant antifungal activity. After taxonomic identification, the strain W0101 was classified into *B. amyloliquefaciens. B*. *amyloliquefaciens* is a gram-positive, endospore-forming, and rod-shaped bacterium, which was identified as a new species of *Baccillus* in 1987 (Priest et al., 1987). In the past few decades, many strains of *B. amyloliquefaciens* have been isolated from soil, plants, food, fresh water, and marine water (Chen et al., 2009; Lee et al., 2016; Boottanun et al., 2017; Cui et al., 2017; Yang et al., 2021). Several isolates of *B. amyloliquefaciens* have exhibited their application potential as plant growth promoters, probiotics, and antibiotics. As effective candidates of biocontrol microorganisms in agriculture, *B. amyloliquefaciens* Pc3, S185, PT14, Rdx5, FZB42, and SWB16 suppressed the growth of many fungal or bacterial pathogens, such as *A. longipes*, *C. gloeosporioides*, and *F. oxysporum* (Chen et al., 2009; Wang et al., 2014; Kim et al., 2015; Ding et al., 2019; Dong et al., 2019; Singh et al., 2021). *B. amyloliquefaciens* W0101 isolated here could suppress 9 pathogenic fungi, such as *A. longipes*, *C. gloeosporioides*, *F. oxysporum*, *S. sclerotiorum*, and *P. amygdali*, showing a broad spectrum of antifungal activity. Thus, *B. amyloliquefaciens* W0101 may be a potential source for the development of biocontrolling agents for plant fungal diseases.

Generally, the antifungal substances produced by *B. amyloliquefaciens* include antifungal proteins, peptides, and small molecular compounds (Li et al., 2021). It has been found that *B. amyloliquefaciens* Pc3 produced 11 lipopeptide compounds and a small molecular compound (isotryptophan) with antifungal activity against *R. solani* (Cui et al., 2017; Ding et al., 2019). *B. amyloliquefaciens* SWB16 also produced fengicin and iturin to suppress the growth of *B. bassiana* (Wang et al., 2014). Moreover, Wang et al. (2002) purified and characterized two antifungal chitinases from *B. amyloliquefaciens* V656, which displayed inhibitory activity on fungal growth. In this study, a 2.4 kDa antifungal peptide, tentatively named W1, was isolated from the fermentation supernatants of the strain W0101. However, the amino acid sequence of W1 only matched with a partial sequence of YajC protein of W0101 by mass spectrometry identification. The sequence analysis showed that W1 was located in the transmembrane helix region of YajC protein, which is a small integral membrane protein with a single transmembrane helix (Fang and Wei, 2011). It is a subunit of the bacterial translocase that transports the majority of secretory proteins across and inserts most membrane proteins into the bacterial cytoplasmic membrane (Fang and Wei, 2011). However, there have not been any reports that the YajC protein is associated with antifungal activity. Thus, we speculated that W1 might be a novel antifungal peptide formed by the proteolysis of YajC protein.

In recent years, many studies have reported the biological activities of peptide fragments derived from physiological precursor proteins (Ciociola et al., 2016). It is currently established that many proteins can release the functional units that are endowed with biological activities different or similar to those of the precursor protein (Ciociola et al., 2016). Many anionic antimicrobial peptides, such as bovine enkelytin and dermcidin, are peptide fragments cleaved from precursor proteins without antimicrobial activity (Harris et al., 2009; Schittek, 2012; Sowa-Jasiłek et al., 2020). A typical example was bovine hemoglobin, which could produce 26 antimicrobial peptides by controlled hydrolysis (Adje et al., 2011). A recent study also reported that a novel peptide CGA-N46 derived from the N-terminus of human Chromogranin A could suppress various *Candida* spp. (Li et al., 2015). In this study, the amino acid sequence of W1 completely matched the part of the N-terminal sequence of YajC protein, indicating that W1 may not be encoded by a bacterial biosynthetic gene of *B. amyloliquefaciens* W0101. We speculated that W1 may be produced by YajC proteolysis, but the details of W1 production need to be further investigated. The three-dimensional structure prediction proposes that W1 is a hydrophobic α-helix peptide with a hydrophilic and cationic arginine residue located at its C-terminal extremity. This amphiphilic structural feature was present in many natural antifungal peptides, such as magainin and cecropins, and made them interact with the lipid bilayer of pathogenic fungal cells (Jin et al., 2005). Thus, these antifungal peptides could inhibit mycelial growth or break the hyphae or spores (Li et al., 2021). It has been found that the α-helix peptide PT14-4a derived from *B. amyloliquefaciens* PT14 causes severe morphological deformation in the conidia and hyphae of *F. solani* and *F. oxysporum* (Kim et al., 2015). The antifungal peptide EP-2, produced by *B. subtilis* E1R-J, can swell and distort the mycelium of the fungi (Wang et al., 2016). Similarly, the SEM observation showed that W1 also ruptured the hyphae of the pathogenic fungi, suppressing the growth of the fungi.

In addition to potent activity, W1 also exhibits remarkable stability of antifungal activity. Its activity remains stable after heating at 100°C–110°C or treatment with solutions of pH 4 and 11. Besides, W1 showed higher tolerance to a variety of proteases against *F. oxysporum*. These characteristics were better than the antifungal peptides produced by *B. amyloliquefaciens* S185, PT14, and *B. velezensis* HNAH 17806 (Kim et al., 2015; Singh et al., 2021; Wang et al., 2021), indicating that W1 has superior application potential in the biocontrol of plant diseases.

## Data Availability Statement

The datasets presented in this study can be found in online repositories. The names of the repository/repositories and accession number(s) can be found below: https://www.ncbi.nlm.nih.gov/genbank/, NZ_CP090477.1.

## Author Contributions

QW performed most of the experiments, analyzed the data, and wrote the manuscript. TH helped with study of antifungal mechanisms and wrote the manuscript. RL help with experimental operations. ZO performed the supplementary SEM observation for manuscript revision. WZ designed the experiments. XC designed the research and revised the manuscript. All authors contributed to the article and approved the submitted version.

## Conflict of Interest

The authors declare that the research was conducted in the absence of any commercial or financial relationships that could be construed as a potential conflict of interest.

## Publisher’s Note

All claims expressed in this article are solely those of the authors and do not necessarily represent those of their affiliated organizations, or those of the publisher, the editors and the reviewers. Any product that may be evaluated in this article, or claim that may be made by its manufacturer, is not guaranteed or endorsed by the publisher.

## Footnotes

1
https://blast.ncbi.nlm.nih.gov/Blast.cgi

2
http://web.expasy.org/protparam/

3
https://novopro.cn/tools/secondary-structure-prediction.html

4
https://mobyle.rpbs.univ-paris-diderot.fr
