# Supplementary material for: Identification of a New Antifungal Peptide W1 From a Marine Bacillus amyloliquefaciens Reveals Its Potential in Controlling Fungal Plant Diseases
Source: Front Microbiol. 2022 Jun 13;13:922454. doi: 10.3389/fmicb.2022.922454 (PMC9237960; doi:10.3389/fmicb.2022.922454)
Supplement: Supplementary file 1 [file Data_Sheet_1.docx]

**Supplementary materials**

**Supplementary** **Figure 1.** **The antifungal activity of the strain W0101 against eight** **pathogenic fungi***.* The letter “A” represents the culture supernatant of W0101, and “B” represents LB medium as control.

**Supplementary** **Figure 2. Chromatographic profile of component Q1 on C18 reverse-phase column by RP-HPLC**. The piperazine buffer (20mM) was used as the blank control. The red box shows the retention time of the unique antifungal peptide of component Q1.

**
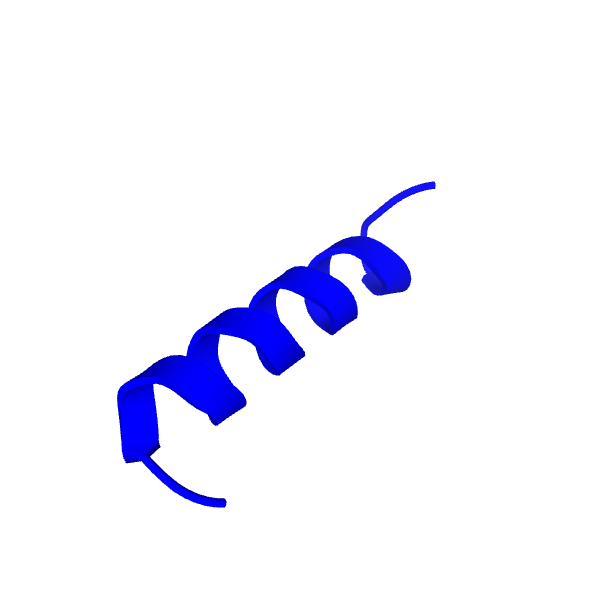
**

**Supplementary** **Figure 3. The predictive three-dimensional structure of W1.** The three-dimensional structure of W1 was predicted using PEP-FOLD3 (https://mobyle.rpbs.univ-paris-diderot.fr).

**Supplementary** **Figure 4.** **Scanning electron microscopy of hyphae and spores of *S. sclerotiorum* treated with W1.** The mycelium of *S. sclerotiorum* was treated with the purified W1 (160 µg/mL) at 30℃ for 24 h, while treated with PBS as control. Images was observed and captured using a JSM-6380LV SEM (JEOL Instruments Inc, Japan) at 15 kv. The white arrows represent the morphological deformation in hyphae and spores when treated with W1.

**Supplementary** **Table 1.** The antifungal activity of strain W0101 against ten phytopathogenic fungi*.*

| Tested fungi | Collection No. | Radius of inhibition zone (mm)* |
| --- | --- | --- |
| *Alternaria longipes* | ACCC 30002 | 34 ± 1.1 |
| *Rhizoctonia solani* Kühn | ACCC 36316 | 30 ± 1.9 |
| *Botryosphaeria dothidea* | ACCC 38026 | 28 ± 0.3 |
| *Trichoderma viride* | ACCC 30902 | 17 ± 0.5 |
| *Alternaria gaisen* | ACCC 37473 | 30 ± 2.1 |
| *Phomopsis amygdali* | ACCC 37078 | 34 ± 1.2 |
| *Fusarium oxysporum* | ACCC 31352 | 28 ± 1.0 |
| *Sclerotinia sclerotiorum* | ACCC 36081 | 17 ± 0.3 |
| *Paecilomyces variotii* | CGMCC 3.776 | 25 ± 1.1 |
| *Colletotrichum gloeosporioides* | ACCC 31200 | 24 ± 0.2 |

*Tests were done in triplicate and the results were shown as mean ± standard deviation (SD) of three experiments.

**Supplementary** **Table 2.** The 16S rRNA gene homology analysis between the strain W0101 and other reference strains from the NCBI GenBank database.

| Strains | Accession No. | Identity% |
| --- | --- | --- |
| *Bacillus velezensis* strain CBMB205 | NR_116240.1 | 99.651 |
| *Bacillus amyloliquefaciens* strain NBRC 15535 | NR_041455.1 | 99.651 |
| *Bacillus amyloliquefaciens* strain MPA 1034 | NR_117946.1 | 99.65 |
| *Bacillus amyloliquefaciens* strain BCRC 11601 | NR_116022.1 | 99.582 |
| *Bacillus siamensis* KCTC 13613 strain PD-A10 | NR_117274.1 | 99.512 |
| *Bacillus subtilis* subsp. subtilis strain 168 | NR_102783.2 | 99.512 |
| *Bacillus nakamurai* strain NRRL B-41091 | NR_151897.1 | 99.512 |
| *Bacillus vallismortis* strain DSM 11031 | NR_024696.1 | 99.442 |
| *Bacillus mojavensis* strain ifo 15718 | NR_118290.1 | 99.43 |
| *Bacillus subtilis* strain DSM 10 | NR_027552.1 | 99.372 |
| *Anoxybacillus sediminis* strain YIM 73012 | NR_174240.1 | 94.302 |
